# High Levels of Aromatic Amino Acids in Gastric Juice during the Early Stages of Gastric Cancer Progression

**DOI:** 10.1371/journal.pone.0049434

**Published:** 2012-11-13

**Authors:** Kai Deng, Sanren Lin, Liya Zhou, Yuan Li, Mo Chen, Yingchun Wang, Yuwen Li

**Affiliations:** Department of Gastroenterology, Peking University Third Hospital, Beijing, China; Pontificia Universidad Catolica de Chile, Faculty of Medicine, Chile

## Abstract

**Background:**

Early-stage gastric cancer is mostly asymptomatic and can easily be missed easily by conventional gastroscopy. Currently, there are no useful biomarkers for the early detection of gastric cancer, and their identification of biomarkers is urgently needed.

**Methods:**

Gastric juice was obtained from 185 subjects that were divided into three groups: non-neoplastic gastric disease (NGD), advanced gastric cancer and early gastric cancer (EGC). The levels of aromatic amino acids in the gastric juice were quantitated using high-performance liquid chromatography.

**Results:**

The median values (25th to 75th percentile) of tyrosine, phenylalanine and tryptophan in the gastric juice were 3.8 (1.7–7.5) µg/ml, 5.3 (2.3–9.9) µg/ml and 1.0 (0.4–2.8) µg/ml in NGD; 19.4 (5.8–72.4) µg/ml, 24.6 (11.5–73.7) µg/ml and 8.3 (2.1–28.0) µg/ml in EGC. Higher levels of tyrosine, phenylalanine and tryptophan in the gastric juice were observed in individuals of EGC groups compared those of the NGD group (NGD vs. EGC, *P*<0.0001). For the detection of EGC, the areas under the receiver operating characteristic curves (AUCs) of each biomarker were as follows: tyrosine, 0.790 [95% confidence interval (CI), 0.703–0.877]; phenylalanine, 0.831 (95% CI, 0.750–0.911); and tryptophan, 0.819 (95% CI, 0.739–0.900). The sensitivity and specificity of phenylalanine were 75.5% and 81.4%, respectively, for detection of EGC. A multiple logistic regression analysis showed that high levels of aromatic amino acids in the gastric juice were associated with gastric cancer (adjusted β coefficients ranged from 1.801 to 4.414, *P*<0.001).

**Conclusion:**

Increased levels of tyrosine, phenylalanine and tryptophan in the gastric juice samples were detected in the early phase of gastric carcinogenesis. Thus, tyrosine, phenylalanine and tryptophan in gastric juice could be used as biomarkers for the early detection of gastric cancer. A gastric juice analysis is an efficient, economical and convenient method for screening early gastric cancer development in the general population.

## Introduction

Gastric cancer is the second most common type of cancer in East Asia, and the prognosis of gastric cancer patients is poor as a result of late detection [1], [2], [3]. To date, stomach cancer survival and patient quality of life is only dramatically improved if the cancer is diagnosed early [4]. Unfortunately, less than 5% of early gastric cancer cases are detected and diagnosed promptly [5].

Endoscopy followed by pathological biopsy is now the preferred method to detect early gastric cancer (EGC). However, EGC is missed frequently during gastroscopy because a minor lesion can be easily overlooked, biopsied inadequately, or interpreted incorrectly by pathologists [6], [7]. EGC is largely asymptomatic prior to the onset of severe symptoms, and an endoscopic investigation based on severe symptoms always delays the detection of stomach cancer. Population-based screening may facilitate the early detection and diagnosis of gastric cancer. However, both the lack of experienced endoscopists and the patient discomfort associated with the procedure reduce the application of this method as a screening tool for EGC.

Less invasive and more efficient biomarkers are needed for the detection of EGC by mass screening. The current biomarkers pepsinogen, carcinoembryonic antigen (CEA), and carbohydrate antigen 19–9 (CA19–9), are not sufficient for accurately predicting gastric cancer at an early stage [8], [9], [10].

It is well established that metabolic abnormalities occur prior to significant morphological changes in malignant tissue [11]. A substantial amount of information concerning the metabolic state of the gastric epithelium is contained in gastric juice. Therefore, gastric juice can be utilized to establish a diagnostic test and identify valuable and efficient biomarkers for EGC screening. In the previous studies, we have established the fluorescence spectrum analysis of gastric juice [12], [13], [14] and identified three fluorescence biomarkers (aromatic amino acids in gastric juice) [15] that can be used to distinguish advanced gastric cancer (AGC) from benign diseases. However, it is still uncertain whether these biomarkers can differentiate between EGC and benign diseases. This study was performed to test the hypothesis that gastric juice biomarkers have diagnostic screening value in the detection of EGC.

**Table 1:** Demographic characteristics of patients.

	NGD (n = 70) Mean±SD	AGC (n = 66) Mean±SD	EGC (n = 49) Mean±SD	*P* c value
Age	58.8±13.4	57.8±14.9	63.8±11.5	0.049
Female/Male	40/30	35/31	16/33	0.023
*Helicobacter pylori* status^a^ (−/+)	44/25	37/25	34/14	0.478
Position				
cardia	–	15	10	
angulus	–	3	13	
fundus	–	3	–	
antrum	–	24	18	
Corpus	–	21	8	
Pathology				
severe dysplasia	–	–	23	
adenocarcinoma	–	49	25	
signet ring cell		17	1	
Lauren's classification^b^				
intestinal type	–	41	24	
diffuse type	–	19	1	
mixed type	–	6	1	

a 6 cases missed;

b 23 cases of severe dysplasia were not included;

c Comparisons were carried out among the three group (NGD, AGC and EGC).

## Methods

### Ethics Statement

The ethics committee of Peking University Health Science Centre approved this research study. Written informed consent was obtained from all participants, and the entire clinical investigation was conducted according to the principles expressed in the Declaration of Helsinki.

**Table 2:** Operating condition for Quantitative HPLC.

					Fluorescence detector		
Time (min)	Flow rate (ml/min)	Column temperature (°C)	A mobile phase^a^ (%)	B mobile phase^b^ (%)	Excitation Wavelength (nm)	Emission Wavelength (nm)	Supplement
5 min	0.8	28	95	5	–	–	Baseline balance
0.0∼10.0	0.8	28	95∼85	5∼15	224	304	Quantitation system
10.0∼14.0	0.8	28	85∼81	15∼19	206	287	
14.0∼15.0	0.8	28	81∼80	19∼20	227	357	
15.0∼23.0	0.8	28	0	100	227	357	
23.0∼30.0	0.8	28	95	5	227	357	

a 0.05% (v/v) trifluoroacetic acid/water;

b pure acetonitrile.

### Sample Collection

Samples were collected at the endoscopy rooms of Peking University Third Hospital. All patient samples obtained for this study were histologically confirmed by mucosal biopsy and/or postoperative pathology from December 2008 to July 2012. After an overnight fast, the patients underwent a gastroscopy. Samples of gastric juice that were free of gross food residue, bile and blood were collected. The samples were separated into 2 ml aliquots and immediately stored at −80°C for subsequent analysis. Warthin-Starry (WS) staining was used to detect the *Helicobacter pylori* infection status in all specimens (The gastric antrum was one of the biopsy sites and the total number of biopsy sites was more than two).

**Table 3:** The measurement of protein and aromatic amino acids in gastric juice.

	NGD (n = 70) Median (P_25_–P_75_)	AGC (n = 66) Median (P_25_–P_75_)	EGC (n = 49) Median (P_25_–P_75_)	*P* a value
Tyrosine (µg/ml)	3.8 (1.7–7.5)	18.3 (6.4–52.3)***	19.4 (5.8–72.4)*** ^b^	<0.0001
Phenylalanine (µg/ml)	5.3 (2.3–9.9)	25.7 (11.1–76.1)***	24.6 (11.5–73.7)*** ^b^	<0.0001
Tryptophan (µg/ml)	1.0 (0.4–2.8)	4.7 (2.5–20.3)***	8.3 (2.1–28.0)*** ^b^	<0.0001
Protein (mg/ml)	1.5 (0.9–2.4)	3.3 (2.3–5.1)***	2.8 (1.6–3.7)*** ^b^	<0.0001

a The three groups (NGD, AGC and EGC) were compared using the Kruskal-Wallis test;

*** Compared with the NGD group using Dunn's test. The *P* value is less than 0.001;

b Comparisons were carried out between AGC and EGC using Dunn's multiple comparison post hoc tests were used. *P* values were calculated (tyrosine, 0.811; phenylalanine, 0.781; tryptophan, 0.691; protein, 0.044 which is less than the significance level of 0.017).

### Participants

Gastric juice samples were obtained from 49 patients with EGC (6 cases were included in a previous publication [15]). There were 26 cases of focal carcinoma and 23 cases of severe dysplasia that could be classified as EGC [16]. In addition to the gastric juice samples isolated from the patients with EGC, samples from 70 patients with non-neoplastic gastric disease (NGD) who underwent an outpatient endoscopy procedure during the same period were used as a control group for comparison. The control group contained 47 patients with chronic superficial gastritis, 18 patients with chronic atrophic gastritis, and 5 patients with peptic ulcers. Additionally, gastric juice samples were randomly collected from 66 patients with AGC (Table 1). There were no significant abnormalities observed in the pre-endoscopic kidney and liver function tests of the recruited subjects. Most of the recruited gastric cancer patients had not been previously diagnosed, and none of the patients showed significant malnutrition (Kamofsky performance status, KPS>90).

**Figure 1:**
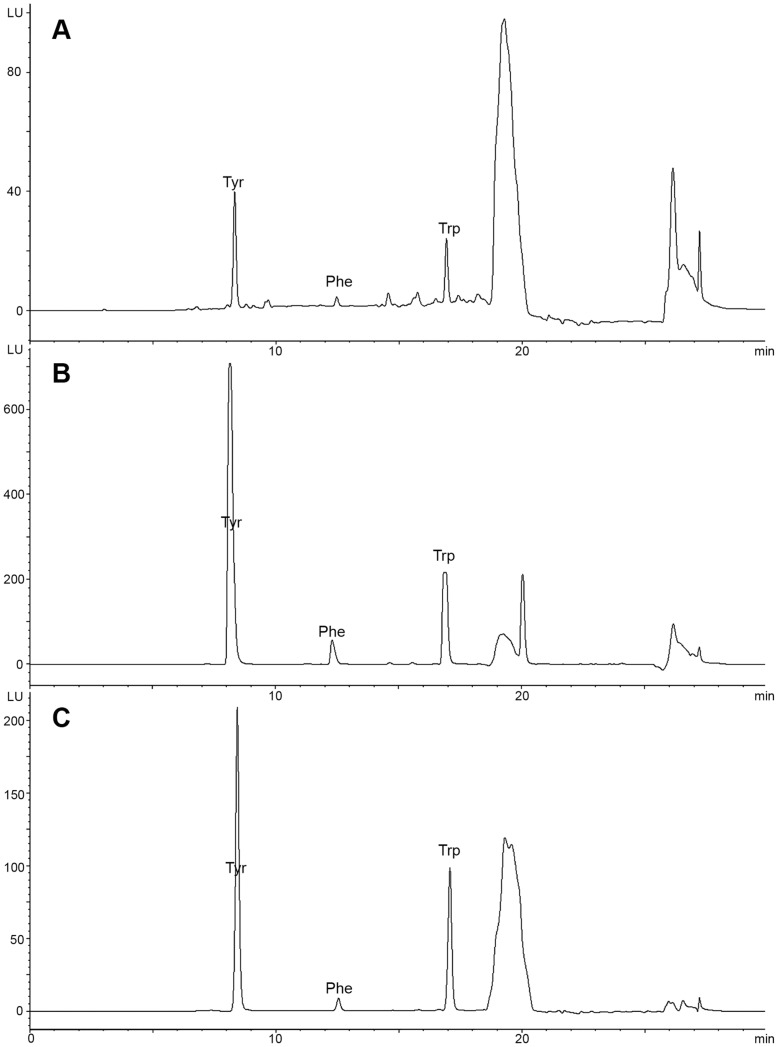
Fluorescence chromatograms of aromatic amino acids in gastric juice. (A) NGD, (B) AGC and (C) EGC group. Tyr, tyrosine; Phe, phenylalanine; Trp, tryptophan.

**Figure 2:**
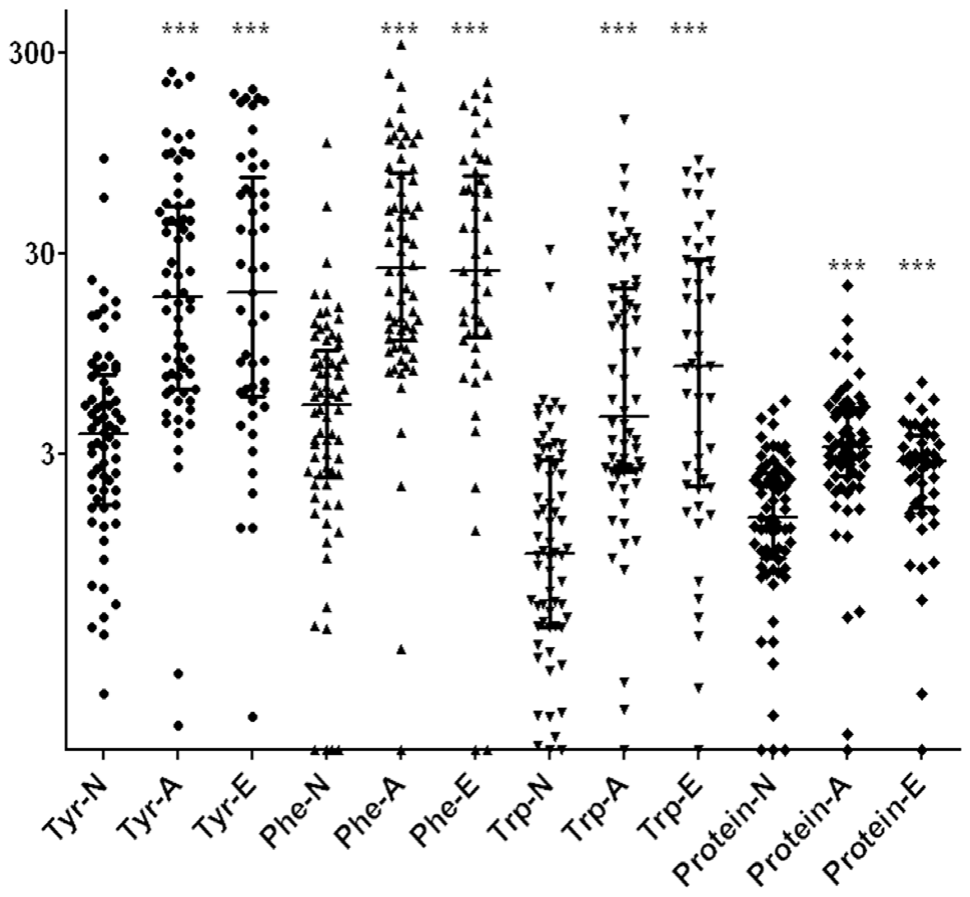
Scatter plot showing the levels of tyrosine, phenylalanine, tryptophan and total protein in gastric juice from NGD, AGC and EGC groups. Tyr-N is the level of tyrosine in gastric juice from the NGD group. Tyr-A is the level of tyrosine in gastric juice from AGC group, and Tyr-E is the level of tyrosine in gastric juice from the EGC group. Phe-N indicates the level of phenylalanine in gastric juice from NGD group. Phe-A indicates the level of phenylalanine in gastric juice from AGC group and Phe-E indicates the level of phenylalanine in gastric juice from EGC group. Trp-N indicates the level of tryptophan in gastric juice from NGD group. Trp-A indicates the level of tryptophan in gastric juice from AGC group and Trp-E indicates the level of tryptophan in gastric juice from EGC group. Protein-N is the level of total protein in gastric juice from NGD group. Protein-A is the level of total protein in gastric juice from AGC group and Protein-E is the level of total protein in gastric juice from EGC group. ***: In comparison with NGD group, *P* value is less than 0.001. The median with interquartile range was shown in figure.

### Sample Treatment

The frozen samples were thawed at room temperature and centrifuged at 14000 rpm for 20 minutes at 4°C. The precipitate was removed, and the upper layer was recovered and temporarily stored at 4°C for the following experiment. The samples were numbered at random. The experimenter was blinded to the diagnosis of the patients recruited to the study.

### Quantification of Total Protein in Gastric Juice

To quantify the protein content, 100 µl of gastric juice supernatant was recovered and immediately diluted with 900 µl of 0.15 M phosphate buffer (pH 7.3) to adjust the pH. The total protein content in the gastric juice was measured using a BCA (bicinchoninic acid) protein assay kit (Beijing Cowin Biotech Co. Ltd., Beijing, China) according to the manufacturer’s protocol. If the protein concentration was out of the working range of the assay (20–2000 µg/ml), the sample was further diluted and reanalyzed.

**Figure 3:**
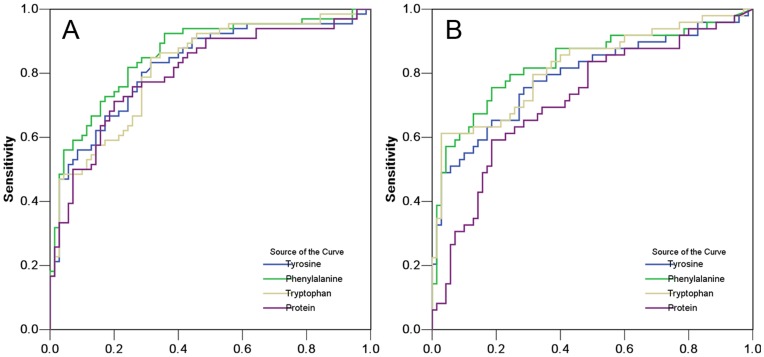
Predictive ability of gastric juice analysis for gastric cancer. (A) ROC curves of tyrosine, phenylalanine, tryptophan and protein in gastric juice for detection of AGC. (B) ROC curves of tyrosine, phenylalanine, tryptophan and protein in gastric juice for detection of EGC.

**Table 4:** Performance of aromatic amino acids for detection of AGC or EGC.

Marker	AUC (SE)	95% CI	Cutoff value	Sensitivity	Specificity	Accuracy
Detection of AGC						
Tyrosine	0.824 (0.036)	0.753–0.895	5.45 µg/ml	83.3% (55/66)	68.6% (48/70)	75.7% (103/136)
Phenylalanine	0.858 (0.033)	0.794–0.922	9.68 µg/ml	81.8% (54/66)	75.7% (53/70)	78.7% (107/136)
Tryptophan	0.818 (0.036)	0.747–0.889	1.94 µg/ml	84.8% (56/66)	68.6% (48/70)	76.5% (104/136)
Protein	0.803 (0.038)	0.728–0.878	2.34 mg/ml	75.8% (50/66)	74.3% (52/70)	75.0% (102/136)
Detection of EGC						
Tyrosine	0.790 (0.045)	0.703–0.877	5.88 µg/ml	75.5% (37/49)	71.4% (50/70)	73.1% (87/119)
Phenylalanine	0.831 (0.041)	0.750–0.911	11.74 µg/ml	75.5% (37/49)	81.4% (57/70)	79.0% (94/119)
Tryptophan	0.819 (0.041)	0.739–0.900	5.67 µg/ml	61.2% (30/49)	97.1% (68/70)	82.4% (98/119)
Protein	0.719 (0.049)	0.623–0.814	2.66 mg/ml	59.2% (29/49)	81.4% (57/70)	72.3% (86/119)

AUC, area under the ROC curve; SE, the standard error of AUC; Cutoff value, optimized cutoff points were the values yielding maximum sums of sensitivity and specificity from the ROC curves; AGC, advanced gastric cancer; EGC, early gastric cancer.

### Quantitative System

Aromatic amino acids in the gastric juice samples were quantitated using a fluorescence detector in conjunction with HPLC [17], [18], and the previously established quantitative system [15] was applied in this study. An HPLC Inlet Filter, a Diamonsil (2) C18 (3 µm, 250×4.6 mm) analytical column (Dikma Technologies, Beijing, China) and an Agilent 1100 series liquid chromatography system (Agilent Technologies, Waldbronn, Germany) were used in this study. The system consisted of a vacuum solvent degassing unit, four solvent gradient pumps, a temperature-regulated automatic sample injector, a column thermostat, and a 3D fluorescence detector (FLD). Chemstation software was used to quantify the amount of aromatic amino acids in the gastric juice samples. Prior to reversed-phase HPLC analysis, the gastric juice supernatant was filtered through a 0.45 µm HPLC filter. The aromatic amino acids in the gastric juice were separated during HPLC on a C18 analytical column using 0.05% (v/v) trifluoroacetic acid/water (HPLC grade; Dikma Technologies) as solvent A and pure acetonitrile (HPLC grade; Merck, Darmstadt, Germany) as solvent B mobile phase modifiers in a linear gradient elution system. After a needle wash, the samples were injected at a volume of 20–100 µl injection volume. The HPLC procedure and experimental conditions for each sample are listed in Table 2.

**Table 5:** Univariate and multivariate analyses of the predictive values of aromatic amino acids for EGC.

	Univariate analysis		Multivariate analysis	
Factor	β Coefficient (SE)^a^	*P* value^b^	Adjusted β Coefficient (SE)^c^	*P* value^d^
Model with tyrosin^e^ included				
Age	0.032 (0.015)	0.036	0.042 (0.018)	0.021
Sex (reference: male)	−1.012 (0.389)	0.009	−1.249 (0.472)	0.008
*Helicobacter pylori* status (reference: negative)	−0.322 (0.404)	0.426	−0.445 (0.486)	0.360
Protein	0.586 (0.161)	<0.001	0.289 (0.189)	0.127
Tyrosine^e^ (reference: <5.88 µg/ml)	2.042 (0.425)	<0.0001	1.801 (0.527)	<0.001
Model with phenylalanine included				
Age	0.032 (0.015)	0.036	0.047 (0.019)	0.016
Sex (reference: male)	−1.012 (0.389)	0.009	−1.179 (0.498)	0.018
*Helicobacter pylori* status (reference: negative)	−0.322 (0.404)	0.426	−0.482 (0.517)	0.351
Protein	0.586 (0.161)	<0.001	0.137 (0.203)	0.498
Phenylalanine^e^ (reference: <11.74 µg/ml)	2.604 (0.453)	<0.0001	2.555 (0.586)	<0.0001
Model with tryptophan included				
Age	0.032 (0.015)	0.036	0.055 (0.022)	0.014
Sex (reference: male)	−1.012 (0.389)	0.009	−0.947 (0.549)	0.085
*Helicobacter pylori* status (reference: negative)	−0.322 (0.404)	0.426	−1.298 (0.696)	0.062
Protein	0.586 (0.161)	<0.001	0.187 (0.209)	0.371
Tryptophan^e^ (reference: <5.67 µg/ml)	3.983 (0.775)	<0.0001	4.414 (0.945)	<0.0001

a Coefficients for each factor were evaluated using univariate logistic regression;

b Two-sided Chi-squared test for coefficients obtained from univariate logisitic regression model;

c Adjusted coefficients for each covariate were evaluated using multivariate logistic regression with adjustment for age, sex, *Helicobacter pylori* status, and protein concentration;

d Two-sided Chi-squared test for coefficients obtained from the multivariate logisitic regression model;

e Because there were collinearities between tyrosine phenylalanine and tryptophan (all R>0.8 ), we assessed the relationship between the levels of tyrosine, phenylalanine or tryptophan and the occurrence of EGC in three separate models, and the adjusted coefficients were estimated in each model.

### Aromatic Amino Acid Quantification

Standard and quality control solutions that contained equivalent concentrations of L-tyrosine, L-phenylalanine and L-tryptophan (99%, Alfa Aesar, Ward Hill, MA, USA) were prepared at concentrations of 0.5, 1.0, 2.0, 5.0, 10.0, 15.0, 18.0 and 20.0 µg/ml to establish calibration curves. The standards were stored at −80°C. Twenty-microliter aliquots of ddH_2_O (blank control solution) and the standard and quality control samples were used to establish and verify the calibration curves before assaying the three aromatic amino acids in the gastric juice samples. If the levels of tyrosine, phenylalanine and/or tryptophan were out of the measured range, the samples were reanalyzed after changing the injection volume (20–100 µl). If a sample concentration was undetectable after several attempts at analysis, it was assigned a score of zero.

### Statistical Methods

When an acceptable normal distribution was achieved, the data were expressed as the means ±SD (standard deviation), and the comparisons among the groups were made using the one-way ANOVA test. When an acceptable normal distribution was not achieved, the data were expressed as the medians (25th to 75th percentile). Kruskal-Wallis tests followed by Dunn's multiple comparison post hoc tests (Bonferroni-corrected significance level of 0.05/3 = 0.017) were used to determine the differences among the three groups. The Kruskal–Wallis and Dunn's tests were performed using Minitab (version 16, Minitab Company, State College, PA) with the KrusMC.MAC macro (obtained from the Minitab website). The Mann-Whitney U test was only used for comparisons between two groups. A Chi-squared test was used to estimate the association between two binary variables. A Pearson correlation was chosen to analyze the association between variables. A partial correlation was used to assess the association between the aromatic amino acid levels and the clinical diagnosis, which resulted in the removal of a set of controlling variables. The receiver operating characteristic (ROC) curves and the areas under the curve (AUC) of the ROC were applied to assess the validity of the biomarkers and to identify the optimized cut-off values. All other statistical tests were two-sided, with *P*<0.05 defined as statistically significant. Logistic regression models were used to compute β coefficients and *P* values for each factor/covariate. All calculations were performed with Excel 2003 (Microsoft Corporation, Seattle, WA, USA), and statistical tests were calculated using SPSS version 15.0 (SPSS, Chicago, IL, USA), SAS 8.1 software (SAS Institute Inc., Cary, NC), and PASS 2008 software package (NCSS, Kaysville, UT).

## Results

### Clinical Characteristics of Patients

This study contained 185 patients separated into 3 groups: 70 NGD patients, 66 AGC patients and 49 EGC patients. There were no significant differences in the occurrence of *Helicobacter pylori* infections (6 cases missed) between the three subgroups. The age and gender varied among the three subgroups (*P* = 0.049 and *P* = 0.023) (Table 1).

### Total Protein in Gastric Juice

The median (25th to 75th percentile) values of the total protein content in the gastric juice samples were as follows: NGD, 1.5 (0.9–2.4) mg/ml; AGC, 3.3 (2.3–5.1) mg/ml; and EGC, 2.8 (1.6–3.7) mg/ml (Table 3). An increase in the total protein content was observed in the gastric juice samples from the AGC and EGC groups relative to that of the NGD group (Dunn's test, AGC vs. NGD, *P*<0.0001; EGC vs. NGD, *P*<0.0001) (Table 1). There was no difference in the total protein content between the AGC group and the EGC group (Dunn's tests, *P* = 0.044, which is greater than the significance level of 0.017) (Table 3).

### Quantitative System

As shown in the fluorescence chromatogram (Figure 1), the three aromatic amino acids were completely separated. The retention times for each of the three amino acids were as follows: tyrosine, 8 min; phenylalanine, 12 min; and tryptophan, 16 min. The calibration curve, linearity and working ranges were as follows: tyrosine, Y = 0.32X+1.30, R^2^ = 0.9999, 0.5–20.0 µg/ml; phenylalanine, Y = 7.15X+1.03, R^2^ = 0.9999, 0.5–20.0 µg/ml; and tryptophan, Y = 0.25X+3.01, R^2^ = 0.9999, 0.5–20.0 µg/ml. The recoveries of tyrosine, phenylalanine and tryptophan were 100.5±2.7%, 101.3±3.4% and 99.6±1.6%, respectively. The precisions of tyrosine, phenylalanine and tryptophan were 100.1±0.5%, 100.0±1.8% and 98.7±1.5%, respectively. This experimental system was stable and reliable for the quantification of aromatic amino acids in gastric juice.

### Aromatic Amino Acids Assay

The median (25th to 75th percentile) values of tyrosine, phenylalanine and tryptophan were 3.8 (1.7–7.5) µg/ml, 5.3 (2.3–9.9) µg/ml and 1.0 (0.4–2.8) µg/ml in the NGD samples; 18.3 (6.4–52.3) µg/ml, 25.7 (11.1–76.1) µg/ml and 4.7 (2.5–20.3) µg/ml in the AGC samples; and 19.4 (5.8–72.4) µg/ml, 24.6 (11.5–73.7) µg/ml and 8.3 (2.1–28.0) µg/ml in the EGC samples (Table 3). The levels of aromatic amino acids in the gastric juice were significantly differently among the NGD, AGC and EGC groups (Kruskal-Wallis test, tyrosine χ^2^ = 49.847, phenylalanine χ^2^ = 62.397, tryptophan χ^2^ = 52.846, all *P*<0.0001). The levels of tyrosine, phenylalanine and tryptophan were significantly increased in the gastric juice obtained from both the AGC and EGC groups compared with those of the NGD group (Dunn's test, AGC vs. NGD, all *P*<0.0001; EGC vs. NGD, all *P*<0.0001) (Table 3 and Figure 2). After adjusting for the effect of the total protein levels in the gastric juice, significant associations still existed between the aromatic amino acid levels and the clinical diagnosis (Partial correlation, AGC vs. NGD: tyrosine, R = 0.334, *P*<0.001; phenylalanine, R = 0.257, *P*<0.001; and tryptophan, R = 0.312, *P*<0.001; EGC vs. NGD: tyrosine, R = 0.378, *P*<0.001; phenylalanine, R = 0.392, *P*<0.001; and tryptophan, R = 0.380, *P*<0.001). There were no significant differences in the levels of tyrosine, phenylalanine and tryptophan between the AGC and EGC gastric juice samples (Dunn's test, *P* = 0.811, *P* = 0.781 and *P* = 0.691, respectively) (Table 3). This result was consistent with previous findings [15] that indicated that no associations exist between *Helicobacter pylori* infection and the levels of aromatic amino acids in gastric juice (Mann-Whitney U test, tyrosine, *P* = 0.871; phenylalanine, *P* = 0.742; tryptophan, *P* = 0.913).

### Validity of Aromatic Amino Acids for the Detection of AGC and EGC

In the ROC curve analysis to distinguish between AGC and NGD, the areas under the curves (AUC) for tyrosine, phenylalanine, tryptophan and total protein were 0.824 [95% confidence interval (CI), 0.753–0.895, *P*<0.001], 0.858 (95% CI, 0.794–0.922, *P*<0.001), 0.818 (95% CI, 0.747–0.889, *P*<0.001) and 0.803 (95% CI, 0.728–0.878, *P*<0.001), respectively (Figure 3A and Table 4). According to the ROC curve analysis to distinguish between EGC and NGD, the AUCs for tyrosine, phenylalanine, tryptophan and total protein were 0.790 (95% CI, 0.703–0.877, *P*<0.001), 0.831 (95% CI, 0.750–0.911, *P*<0.001), 0.819 (95% CI, 0.739–0.900, *P*<0.001) and 0.719 (95% CI, 0.623–0.814, *P*<0.001), respectively (Figure 3B and Table 4). The optimized cut-off values to distinguish AGC or EGC from NGD were chosen by determining the maximum sums of sensitivity and specificity from the ROC curves (Table 4). After selecting the optimized values, the sensitivity, specificity and accuracy needed to distinguish between AGC and NGD were as follows: tyrosine, 83.3%, 68.6% and 75.7%; phenylalanine, 81.8%, 75.7% and 78.7%; tryptophan, 84.8%, 68.6% and 76.5%; and total protein, 75.8%, 74.3% and 75.0%. After selecting the optimized values, the sensitivity, specificity and accuracy needed to distinguish between EGC and NGD were as follows: tyrosine, 75.5%, 71.4% and 73.1%; phenylalanine, 75.5%, 81.4% and 79.0%; tryptophan, 61.2%, 97.1% and 82.4%; and total protein, 59.2%, 81.4% and 72.3% (Table 4). After selecting the optimized values reported in our previous study [12], [15], the sensitivity, specificity and accuracy for the detection of EGC were as follows: tyrosine, 51.0%, 94.3% and 76.5%; phenylalanine, 79.6%, 74.3% and 76.5%; tryptophan, 61.2%, 94.3% and 80.7%; and total protein, 83.7%, 62.9% and 71.4%. Using a different optimized value, we obtained similar results. Using a combination of tyrosine, phenylalanine and tryptophan did not significantly improve the detection of AGC or EGC because the concentrations of the three aromatic amino acids in the gastric juice samples were closely related to each other (tyrosine-phenylalanine, R = 0.871; tyrosine-tryptophan, R = 0.941; phenylalanine-tryptophan, R = 0.840, *P*<0.001 for each).

### Logistic Regression Analysis

In the logistic regression analysis, the association between the levels of aromatic amino acids in the gastric juice and EGC was evaluated. Univariate logistic regression analyses were performed to evaluate the predictive values of candidate biomarkers for the detection of EGC. In these univariate analyses, the β coefficients (SE) of age, sex, *Helicobacter pylori* status, protein and elevated aromatic amino acid levels in gastric juice were calculated. Four variables (age and elevated tyrosine, phenylalanine and tryptophan levels in gastric juice) had a *P*<0.05 and were identified using multiple logistic regression analysis (Table 5). Because there were collinearities between tyrosine, phenylalanine and tryptophan (all R>0.8), we assessed the relationship between the levels of tyrosine, phenylalanine or tryptophan and EGC in three separate models and estimated the adjusted coefficients in each model. The elevated levels of these aromatic amino acids in the gastric juice were associated with EGC (tyrosine: adjusted β coefficient 1.801, SE 0.527; phenylalanine: adjusted β coefficient 2.555, SE 0.586; tryptophan: adjusted β coefficient 4.414, SE 0.945) (Table 5). Thus, elevated levels of aromatic amino acids in the gastric juice may be independent markers for the detection of EGC.

## Discussion

In our previous studies, we have observed increased fluorescence of gastric juice in AGC and identified three fluorescence biomarkers for screening advanced gastric cancer [12], [15]. Whether these potential biomarkers are useful for the early detection of gastric cancer is uncertain. In our current investigation, we tested the validity of using aromatic amino acids in the gastric juice for the diagnosis of EGC. Our results show that high levels of aromatic amino acids are present in the gastric juice from patients with EGC compared to NGD patients. Thus, high levels of aromatic amino acids in the gastric juice may be valuable for early detection of EGC.

Because of the thesmall sample size, power analysis for Kruskal-Wallis test and ROC AUC were performed. To our knowledge, the formulas or procedures for determining the sample size necessary to test for differences in multiple-sample locations using nonparametric methods are much more complicated [19], and no power or sample size calculation method is currently available regarding the generic alternative hypothesis for the Kruskal-Wallis test [20]. Lachenbruch and Clements suggested that the Kruskal-Wallis test might have greater power than the F-test when the population distributions are not normal. They claimed that, compared with the F-test, the Kruskal-Wallis test was more robust against departures from the assumption of the equality of variance [21]. Thus, using the power of the F-test to approximate the power of the Kruskal-Wallis test may provide a poor lower bound for normal distributions. Following a rank transformation, the proximate powers (1 - β error) were calculated using the F-test (ANOVA) as implemented in SAS 8.1. All of the proximate powers are more than 0.99. Thus, we can reliably conclude that the levels of total protein and aromatic amino acids in gastric juice are significantly different among NGD, AGC and EGC. Power analyses for the ROC curves were performed using PASS 2008 software. None of the powers was less than 0.99. Therefore, it is acceptable to conclude that high levels of aromatic amino acids in the gastric juice may be valuable predictors for AGC or EGC.

The enhanced production of aromatic amino acids in EGC corresponds to the results for a previous study [15] and is consistent with early reports in other malignant diseases (blood [22], lung [23], [24], [25], breast [23], [24], bladder [26], etc.). We speculate that the following reasons may account for this phenomenon. First, tumour cells require sufficient aromatic amino acids to accumulate in the cancer foci to meet the sharply increased needs for protein synthesis to support rapid growth. A high expression of amino acids transporters (such as L-type amino-acid transporter 1) in various tumours [27], [28], [29], [30], [31], [32], [33], [34], [35] has been reported in many studies. Amino acid transporters may enhance the transport of amino acids and accumulate aromatic amino acids near cancer foci. The infiltration of excessive aromatic amino acids may significantly increase in gastric juice in EGC compared to NGD. Secondly, aberrant metabolism in cancer cells would precede the visible changes in stomach carcinogenesis [11]. It is known that the abnormalities of amino acid transport and metabolism in a cancerous region occur frequently in the early stage of gastric cancer. Gastric juice, which is generated by the gastric epithelium, contains a substantial amount of information about the metabolic status of the epithelium. Therefore, the information obtained from analysing the gastric juice may be useful in separating biomarkers for the early detection of gastric cancer. Finally, matrix metalloproteinases (MMPs), which are enzymes that degrade the extracellular matrix and basement membrane, play roles in the early phase of tumourigenesis [36], [37], [38], [39], [40]. The over-expression and activity of MMPs accelerates the degradation of extracellular matrix and basement membrane. The degradation of matrix generates aromatic amino acids in the gastric juice surrounding tumour cells. Additionally, blood aromatic amino acids could easily penetrate the impaired basement membrane and epithelia of carcinoma foci and enhance amino acid levels in gastric juice. These causes could explain the significant enhancement of aromatic amino acids in gastric juice from EGC patients.

The early phase of gastric cancer has few symptoms and can be easily missed by conventional endoscopy [6], [41]. These problems reduce the rate of early detection and effective treatment for gastric cancer. Abnormal levels of aromatic amino acids in the gastric juice may be used to monitor the occurrence of amino acid metabolism disorders and reveal early malignant transformations that occur in the stomach. Notably, our findings indicate that the levels of aromatic amino acids in the gastric juice can distinguish EGC from gastritis. This finding introduces the possibility that these biomarkers can be used for the mass screening of gastric cancer at an early stage. The measurement of aromatic amino acid levels in gastric juice samples may be an efficient, economical, rapid, and convenient method for detecting AGC or EGC in a population.

Although aromatic amino acids may be promising biomarkers for EGC, there are still drawbacks associated with this approach. The use of less invasive procedures would be beneficial for screening purposes. The gastric juices in our study were aspirated during endoscopy, which would severely limit the applicability of assessing gastric juice biomarkers for the detection of EGC. To increase the potential application of this finding, non-endoscopic means can be introduced for sampling gastric juice. An endogastric capsule [42], [43], [44] is a 45–50 cm length of nylon thread connected to a capsule contained absorbent paper. A capsule can make gastric juice sampling more acceptable, painless and convenient for patients compared to gastric juice collection by endoscopy. The levels of aromatic amino acids in the gastric juice can be influenced by the fluid and food consumed by the patient. Therefore, full fasting in patients with poor gastric motility is essential for enhancing the accuracy of biomarker assays and gastric cancer detection. Because the sample size in this study is small, larger studies are required to determine whether aromatic amino acids in gastric juice can help in screening early-stage gastric cancer. Additionally, further studies will identify the optimized boundary values for each marker. Once the diagnostic screening values are identified, new methods (such as mass spectrometry) could allow for the simple, rapid and high-throughput detection of aromatic amino acids in gastric juice.

In conclusion, significantly increased levels of aromatic amino acids were found in gastric juice from patients with late-stage or early-stage gastric cancer compared to the NGD group. An analysis of aromatic amino acids may be an efficient method for the early detection of gastric cancer. If validated in a large-scale study, aromatic amino acids in gastric juice would be useful as biomarkers for screening EGC in the general population.
